# New Challenges in Bladder Cancer Diagnosis: How Biosensing Tools Can Lead to Population Screening Opportunities

**DOI:** 10.3390/s24247873

**Published:** 2024-12-10

**Authors:** Fabiana Tortora, Antonella Guastaferro, Simona Barbato, Ferdinando Febbraio, Amelia Cimmino

**Affiliations:** 1 Institute of Genetics and Biophysics “A. Buzzati Traverso”, National Research Council (CNR), 80131 Naples, Italy; fabiana.tortora@igb.cnr.it (F.T.); antonella.guastaferro@igb.cnr.it (A.G.); simona.barbato@igb.cnr.it (S.B.); amelia.cimmino@igb.cnr.it (A.C.); 2 Institute of Biochemistry and Cell Biology, National Research Council (CNR), 80131 Naples, Italy

**Keywords:** bladder cancer diagnosis, population screening, biomarkers, lncRNA

## Abstract

Bladder cancer is one of the most common cancers worldwide. Despite its high incidence, cystoscopy remains the currently used diagnostic gold standard, although it is invasive, expensive and has low sensitivity. As a result, the cancer diagnosis is mostly late, as it occurs following the presence of hematuria in urine, and population screening is not allowed. It would therefore be desirable to be able to act promptly in the early stage of the disease with the aid of biosensing. The use of devices/tools based on genetic assessments would be of great help in this field. However, the genetic differences between populations do not allow accurate analysis in the context of population screening. Current research is directed towards the discovery of universal biomarkers present in urine with the aim of providing an approach based on a non-invasive, easy-to-perform, rapid, and accurate test that can be widely used in clinical practice for the early diagnosis and follow-up of bladder cancer. An efficient biosensing device may have a disruptive impact in terms of patient health and disease management, contributing to a decrease in mortality rate, as well as easing the social and economic burden on the national healthcare system. Considering the advantage of accessing population screening for early diagnosis of cancer, the main challenges and future perspectives are critically discussed to address the research towards the selection of suitable biomarkers for the development of a very sensitive biosensor for bladder cancer.

## 1. Introduction

**Figure 1:**
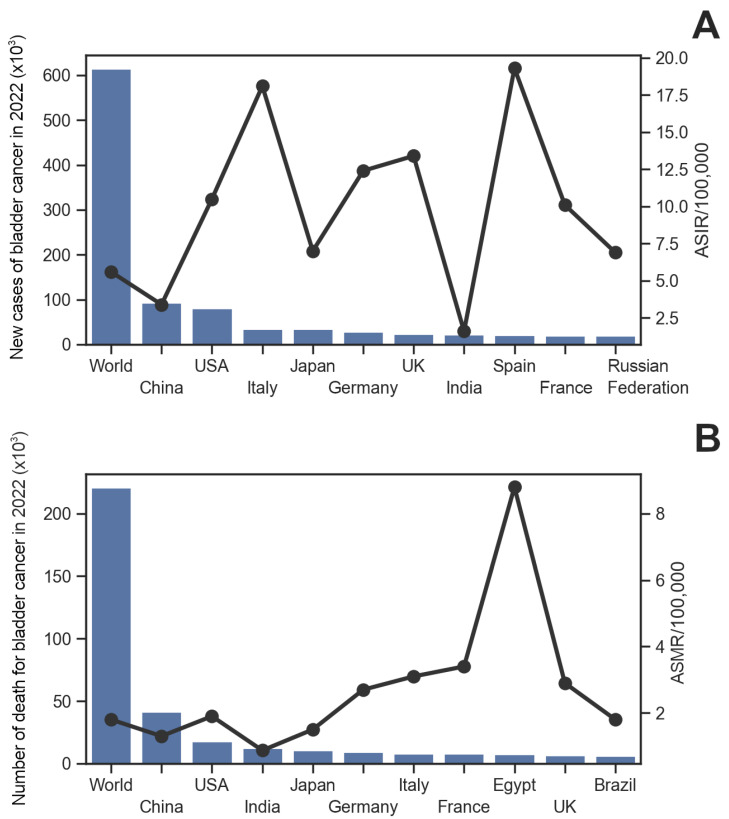
Bar plot of the global and national incidence/mortality of bladder cancer in 2022. The 10 countries with the highest number of bladder cancer cases (**A**) and the highest number of deaths from bladder cancer (**B**) are reported. The age-standardized rate for incidence/mortality (black dots) is expressed as the number of events per 100,000 people. Data from GLOBOCAN 2022 [2,4].

Bladder cancer (BlCa) remains the most common malignancy of the urinary tract and the 9th most common cancer worldwide. Its incidence is higher in men (6th most common cancer) than in women (17th most common cancer). In 2020, more than 500,000 people were diagnosed with BlCa worldwide, and more than 200,000 individuals died from the disease [1]; these numbers rose in 2022 to more than 600,000 and 220,000, respectively [2]. Although the age-standardized incidence rate (ASIR) varies widely across geographic regions, it is expected to continue to increase over the next decade [3], while the age-standardized mortality rate (ASMR) has begun to decline in developed countries and increasing in low-income regions of the world [2] (Figure 1).

In addition to geographic location and age, several risk factors for BlCa have been identified; however, the risk varies between sexes and is strongly influenced by exposure to multiple carcinogens, of which smoking is the most common [5]. The main symptom of BlCa is microscopic or gross hematuria. When bladder tumors are diagnosed, about 75% of cases are diagnosed as urothelial BlCa confined to the mucosa (NMIBlCa—non-muscle invasive disease) [6]. In the remaining 25–30% of patients, BlCa has penetrated the deeper layers of the bladder wall (MIBlCa—muscle invasive disease) or has formed metastases. Transurethral resection of bladder tumors (TURBT) is the mainstay of treatment for patients with NMIBlCa, while patients with MIBlCa undergo radical cystectomy. To prevent recurrence and progression, TURBT is supplemented with intravesical instillations in selected patients. Despite many advances in surgery and anesthesia and the widespread use of perioperative chemotherapy, long-term survival rates for patients with urothelial BlCa have remained unchanged for decades [7].

**Figure 2:**
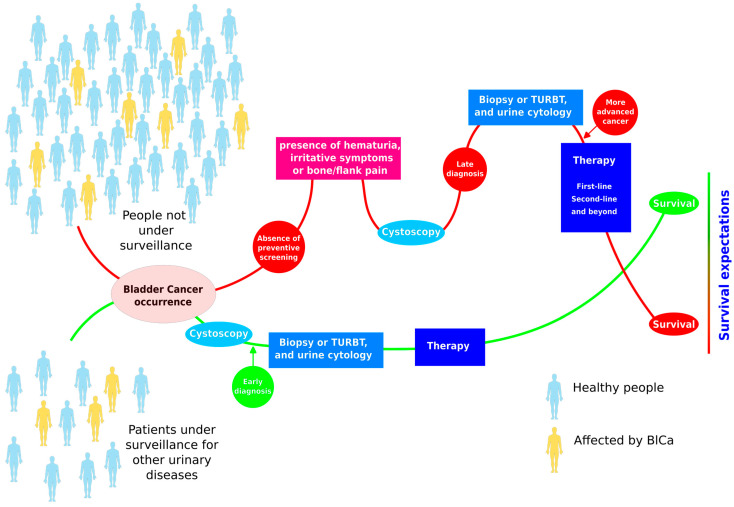
The advantages of surveillance for bladder cancer can reduce the risk of adverse outcomes leading to death.

One of the major limitations of BlCa management is the lack of prevention due to the absence of tests that allow its prediction. In fact, there is no universally accepted screening program for BlCa. This may be due to the low incidence of the invasive form of the disease and the lack of optimal screening tools. However, screening is thought to be effective in carefully selected populations. In individuals known to have aristolochic acid nephropathy who underwent cystoscopy every two years for 10 years, half were diagnosed with BlCa. After a mean follow-up of 94 months, no one died from the disease [8]. Therefore, it is important to identify high-risk subgroups that should be included in appropriate screening programs, in support of the idea that screening is essential to protect against BlCa adverse events (Figure 2).

Although cystoscopy is considered the gold standard for diagnosing BlCa, from a diagnostic perspective, it cannot be used for early screening of the disease because of the invasiveness and the pain experienced by most patients in the procedure. In fact, no one would undergo cystoscopy for preventive reasons and in the absence of symptoms. Also, although technological advancements, such as optical (fluorescent) cystoscopy, have enhanced the identification and analysis of BlCa, poor sensitivity in detecting early-stage tumors remains a significant obstacle for cystoscopy procedures. Moreover, due to its high recurrence rate and requirement for regular cystoscopies during surveillance, BlCa is one of the most expensive cancers to treat.

## 2. Challenges

### 2.1. Diagnostic Tools for Bladder Cancer Alternative to Cystoscopy

Urinary cytology is already used for BlCa diagnosis and follow-up; however, despite the advantage of being a non-invasive test, its sensitivity for diagnosing BlCa is too low (54%) to be proposed to substitute cystoscopy [9]. In particular, it is more sensitive in high-grade tumors (79%) and less sensitive in low-grade ones (16%) [10,11,12]. Additionally, urine cytology is user-dependent, and its low cellular yield, urinary tract infections, and/or stones can hinder the examination. Also, there are now more concerns than ever before about the low sensitivity of cytology because of the non-negligible percentage of rare variants that are frequently even harder to diagnose and the ambiguous flat lesions reported during cystoscopies [13,14,15]. As a result, BlCa cannot be ruled out by a negative cytology and a positive outcome, always require the identification of the bladder lesion following a cystoscopy to determine the final diagnosis.

These challenges led researchers to focus on developing alternative, non-invasive, inexpensive, labor-intensive, non-observer-dependent, quick, and accurate detection tests on urine. Hence, a new generation of non-invasive genomic, epigenetic, transcriptomic, and morphological information from exfoliated cells was successfully enabled. The advantage of biomarkers is their simplicity in the method of clinical endpoint measurement and their reproducibility, along with repeated, immediate analysis and cost-effectiveness when compared to other diagnostic and staging tools [16,17].

The goal of current research is to identify a biomarker with a high negative predictive value (NPV) that can definitively rule out the presence of the tumor because failing to do so could have catastrophic consequences for the patient. Each biomarker category has pros and cons of its own. For instance, protein biomarkers are less expensive than genomic ones, but they appear to be less accurate. Other prospective clinical trials are required to confirm the data, so it is difficult to implement their potential in clinical practice. However, the considerable BlCa heterogeneity, including intra-tumoral heterogeneity as well as interpatient variability, implies that even the most accurate genetic testing may fail to detect a disease based on the exfoliated cells in urine samples [18,19].

In fact, although some of the urine-based biomarkers were granted U.S. Food and Drug Administration (FDA) approvals, their influence in clinical practice is still limited. A high NPV is the most crucial characteristic a new biomarker should possess. The acceptable NPV should not depend on the operator and should be very near 100%. Panels of biomarkers may be an alternative to increase the sensitivity of the tests for BlCa diagnosis, as well as combining the most mutated genes with common epigenetic modifications, or tumor environmental factors, like immunological response, with tumor-induced metabolic alterations. Moreover, it is critical that the test should be affordable and easily accessible, even in the modern urology practice, which involves the use of telemedicine.

As a result, the clinical use of FDA-approved tests, such as protein-based biomarkers (BTA-TRAK, BTA-STAT, and NMP22), genetic-based biomarkers (EpiCheck) and cell-based markers (UroVysion, uCyt+, or ImmunoCyt) [9,20,21] (Table 1), recognized and identified as the most frequently used markers in patient’s surveillance and diagnosis, has decreased recently due to a lack of clear indications for their use, their high cost, their complex processing, or even availability issues.

### 2.2. How Biosensing Tools Can Change Bladder Cancer Diagnosis 

Common methods for identifying cancer biomarkers are typically met with a great deal of criticism because they involve sizable, complex, and costly equipment that needs to be operated by skilled personnel in specialized labs and takes a long time to complete [32]. Therefore, developing new strategies to get over these barriers is essential for the predictive and early detection diagnosis of disease. Improving the prognosis and health of cancer patients also depends on the quick and precise identification of several biomarkers at different molecular levels [33].

Biosensors offer a viable answer to the aforementioned issues in this area. They are non-invasive devices exhibiting extreme sensitivity and selectivity for specific biomarkers [34]. With the aid of a transducer, these devices transform the signals detected by a bioreceptor or biorecognition element into quantifiable signals [35]. They are distinguished by their affordable costs, rapid evaluation, efficient shipping, precise measurement, and the ability to find biomarkers for tumor cells even in the absence of symptoms [36]. Enzyme, nucleic acid, aptamer, antibody, and mixed recognition element-based biosensors are the main categories based on the bio-recognition element [37]. Prior to each use, most sensors require calibration, and some sensors are limited to detecting the analytes at specific concentration ranges [38]. Biosensor efficiency is further increased by the bioreceptor’s stability because bioreceptors can be sensitive to variations in temperature, pressure, and pH level. Consequently, the long-term preservation and use of bioreceptors present significant challenges. Any changes to the typical environmental conditions, such as temperature or pH variations, could lead to a reduction in the capacity for sensing. Given that environmental circumstances change over time, biosensors with a larger stability range are significantly preferable [39].

Literature suggests a growing tendency to use multiple analytes, such as biomarker panels or combinations of biomarkers with clinical variables, to confirm the presence of malignant cells [40,41]. Therefore, the multi-sensing capacity of the biosensor must be improved to achieve higher specificity [42].

The detection of various analytes and cost reduction are facilitated by the combination of 3D printing [43] and microfluidic technologies [44,45]. The World Health Organization (WHO) states that to meet the demands of the public, we should have ASSURED (A cheap, Sensitive, Specific, User-friendly, Robust and speedy, Equipment-free, and given to people in need) Devices [46]. On the other hand, commercial and clinical research interests in bladder cancer were redirected toward the urine-secreted biomarkers owing to the continuous contact of urine with tumor tissue.

Few solutions are now available or under development using biosensing devices for BlCa diagnosis, with organic compounds, proteins, nucleic acids, and cancer cells as main targets in the urine samples. Biosensors for cancer cell detection typically utilize photo-specific identification techniques, leveraging the fluorescence of Hexaminolevulinate-induced Protoporphyrin IX. This approach enables the detection of immuno-captured bladder cancer cells by targeting their Epithelial Cell Adhesion Molecule (EpCAM) through the use of specific anti-EpCAM antibodies [47,48].

A portable diagnostic device has been developed using environmentally sensitive fluorophores, namely Nile Red, Eosin Y, and Rose Bengal, as sensing agents to detect volatile organic compounds specifically present in the urine samples of BlCa patients [49]. Electrochemical ELISA-based immunosensors are commonly used for the detection of protein biomarkers in bladder cancer, such as nuclear mitotic apparatus protein 1 (NUMA1) and complement factor H-related 1 (CFHR1) [50,51,52]. In particular, potential-resolved electrochemiluminescence has garnered significant attention for the detection of multiple tumor biomarkers thanks to its rapid response, high sensitivity, and low background signal [52]. Surface plasmon resonance imaging biosensors have also been employed for detecting various BlCa protein biomarkers, including the concentration of transmembrane protein podoplanin in urine [53] and the levels of collagen IV, laminin-5, and fibronectin in blood serum [54]. Recently, significant efforts have focused on developing biosensing devices for detecting nucleic acids in urine samples to diagnose BlCa, with particular emphasis on identifying microRNAs (miRNAs) and circular RNAs (circRNAs). RNA molecules can be stabilized in urine samples by using specific preservatives, such as RNA Later, which inhibits ribonucleases and preserves the RNA quality for longer periods. An electrochemical biosensor has been developed for the highly sensitive and specific detection of miRNA-21 in human urine, aimed at diagnosing and classifying BlCa. This biosensor is based on single-stranded DNA functionalized single-walled carbon nanotubes, which enhance the detection performance [55]. Also, a bimodal waveguide biosensor, an innovative common-path interferometric sensor based on the evanescent field detection principle, has been developed for detecting miRNA-181a in urine liquid biopsies, enabling the diagnosis of BlCa [56]. Finally, Cheng et al. developed an electrochemical biosensor to detect femtomolar concentrations of circRNA using a CRISPR/Cas13a system specifically engineered to target the characteristic back-splice junction site of circRNA. Upon activation by circRNA, the CRISPR/Cas13a system cleaves uracil bases within a DNA tetrahedron immobilized on a gold electrode’s surface. This cleavage disrupts the DNA tetrahedron structure, releasing the electrochemically active molecule methylene blue [57].

Because of its immense potential for illness detection and personal health management, portable point-of-care testing, or POC, is currently receiving a lot of attention. POC tests have the inherent benefits of affordability, quick response, and simplicity in miniaturization and integration. However, the low quantity of disease-relevant biomolecules in highly complex biological samples results in lower detection sensitivity, which hinders the practical application of biosensors-based POC [55]. The only currently commercially available test is NMP22 BladderChek, which is based on a POC solution using a chromatographic immunoassay for the qualitative detection of the nuclear matrix protein 22 (NMP22) protein, which is released by bladder cancer cells at higher levels than normal cells [58,59]. However, the specificity and sensitivity of this tool are lacking, reaching 99% sensitivity and NPV only when combined with cystoscopy [60,61]; therefore, it is still far from being proposed as an alternative to cystoscopy in clinical practice. Over the past decade, several NMP22 biosensors have been developed and proposed. Among the most promising options for its detection are colorimetric, electrochemical, and fluorescence-based biosensors [59,62]. These sensors offer improved detection sensitivity and address the limitations of current diagnostic methods; however, they still exhibit deficiencies that limit their potential clinical use.

### 2.3. Artificial Intelligence Role in the Bladder Cancer Diagnosis

Since the development of computers and software, artificial intelligence (AI) has been studied in relation to the human brain’s capacity to learn from experience, quickly adapt to new situations, create and work with abstract concepts, and affect the environment. AI has become a widely discussed topic, not just in academic and scientific contexts. Its potential to interact with everyday activities like social media, smart devices, driving, and chat conversations with AI software has us all spellbound [63,64]. In the medical sciences, AI applications are helping to increase the possibility of diagnosing BlCa. In searching for an improvement in BlCa diagnosis and an alternative to invasive cystoscopy, there have been increased attempts in the past several years to train AI-based systems for the examination of bladder tissue and urine samples. AI tools for the diagnosis of BlCa combine imaging with cystoscopy-based tumor identification, tumor staging, and tumor grading [5]. Regarding the diagnosis, prognosis, and outcome prediction of BlCa, both AI subsets represented by machine learning (ML) and deep learning (DL) techniques are extensively researched [65,66,67]. Urine cytology’s diagnostic accuracy has been enhanced by using ML and DL techniques, particularly for low-grade malignancies, by automatically identifying atypical and BlCa cells [68,69,70,71,72,73,74]. Two studies have also examined the application of AI-based technologies in this competition to evaluate urine metabolomes and find biomarkers that may be connected to BlCa [75,76]. Notably, AI algorithms demonstrated higher accuracy when analyzing cystoscopy data; they often demonstrated more accuracy for diagnosing BlCa than when they were used to analyze urine samples. The investigation of AI methods applied to imaging modalities such as CT and MRI scans for BlCa initial diagnosis is still limited compared to other urological malignancies (e.g., kidney and prostate cancers) because of the previously mentioned pivotal role of endoscopy for the detection of BlCa [77,78,79,80,81,82]. Another important development for the near future is the potential for using deep learning and machine learning algorithms to discover innovative medications, treatment plans, and biomarkers. However, there are still several obstacles to be addressed, including data quality, universal application, and ethical considerations. More research is needed to provide more reliable information on this exciting technology’s place in the bladder cancer diagnosis and treatment process.

### 2.4. Social, Economic and Educational Aspects in Bladder Cancer Management

Despite significant advancements in medical care, substantial global health disparities persist that present serious obstacles to public health [83]. Socioeconomic status (SES), or a person’s place in society, is a major factor in many of these inequities [84]. Socioeconomic determinants of health, like income, education, and/or occupation status, are frequently used to measure SES. People with lower levels of social determinants are often more likely to experience poorer health and health outcomes [85].

Heart disease [86], lung disease [87], poor mental health [88], cancer [89], and numerous other diseases [90] are among the many illnesses for which there is a wealth of evidence supporting this relationship. Most notably, there is a correlation between a lower SES and a higher death rate from all causes [91]. These variations in health occur both within nations, such as Canada, and between nations with differing degrees of development [92].

According to a review paper, BlCa is most common in North America and Europe, two of the world’s most developed countries, although it is also seen in Northern Africa and Western Asia. This is most likely because of infections with Schistosoma haematobium [93]. According to a retrospective study conducted in the United States, the prognosis of bladder cancer is directly impacted negatively by a lower SES (as determined by the income and education quintiles) [94]. According to a recent study on economic inequality, those in the lowest income quartile had a higher lifetime likelihood of developing bladder cancer and having a poorer prognosis [95]. According to a different study, people with the lowest levels of education were twice as likely to acquire bladder cancer as people with the highest levels of education [96].

Knowing how bladder cancer is distributed within socioeconomic categories might help policymakers and healthcare providers make more informed decisions about which patient populations may need more attention in terms of therapy and/or preventive care.

### 2.5. Future Perspective Ultraconserved Regions in Human Genome as Highly Sensitive Biomarkers for Early Diagnosis

The advent of next-generation sequencing and gene expression profiling has facilitated the exploration of molecular biomarkers with prognostic and predictive value in bladder cancer [97]. Among these, long non-coding RNAs (lncRNAs) have emerged as significant contributors to the prediction of clinical outcomes [98]. Despite this, limited research has been conducted on developing prognostic models specifically based on lncRNA expression. Identifying a distinct lncRNA signature is therefore crucial to guide future targeted therapies and improve the prognosis of bladder cancer patients.

The family of transcribed ultraconserved regions (T-UCRs) comprises 481 lncRNA transcripts, each approximately 200 nucleotides in length, which are completely conserved across the genomes of rats, mice, and humans [99]. While most lncRNAs exhibit relatively low conservation rates (30–40%), the remarkable evolutionary conservation of T-UCRs suggests a higher degree of regulatory stringency and responsiveness to biological changes. This conservation, often viewed as an indicator of functional significance, highlights their potential as reliable biomarkers for population-level screening in human diseases such as cancer [100]. However, RNA sequencing (RNA-seq) datasets are not suitable for analyzing T-UCRs, as many of these sequences are no longer represented in current databases. Furthermore, research on T-UCRs remains in its early stages, and substantial work is required to fully elucidate their biological functions. To date, dysregulation of T-UCRs has been associated with several human diseases, including neurological, cardiovascular, and developmental disorders [101,102]. Notably, modulating T-UCR expression levels holds potential for cancer therapies, as their relative expression can reflect the stage and progression of the disease [103,104].

Recent studies on bladder cancer tissues have reported altered expression levels and subcellular localization of specific T-UCRs. Based on these findings, we hypothesize that a subset of T-UCRs may serve as critical indicators of BlCa status, offering valuable insights into disease diagnosis and progression [105]. In particular, the most dysregulated T-UCR identified in BlCa is uc.8+ lncRNA, which exhibits a more than sixfold increase in expression levels [101]. Up to now, in the T-UCRs literature, the uc.8+ alteration has not been reported in other types of cancers, suggesting that its upregulation is exclusive of BlCa samples [100]. Moreover, experimental evidence suggested early alteration of uc.8+ expression in BlCa development for both high- and low-grade cancer [101]. This significant upregulation highlights uc.8+ as a potential key player in the pathophysiology of bladder cancer, warranting further investigation into its role and potential as a diagnostic or therapeutic target [101,106].

To date, only a limited number of studies have applied machine learning techniques to utilize T-UCRs as biomarkers, aiming to harness data and improve performance in the early diagnosis of bladder cancer. A recent study identified a panel of T-UCRs, including uc.8+, that can reliably distinguish between healthy individuals and bladder cancer patients, achieving 100% perfect classification [105]. Despite the low number of samples analyzed, these results highlight that the selected T-UCRs permit reliable and early predictions of BlCa. The identification of panels comprising validated markers associated with clinical and pathological variables may represent the most promising approach for precise risk stratification and informed clinical decision-making despite the differing molecular pathogenesis of invasive and non-invasive bladder cancer.

## 3. Conclusions

**Figure 3:**
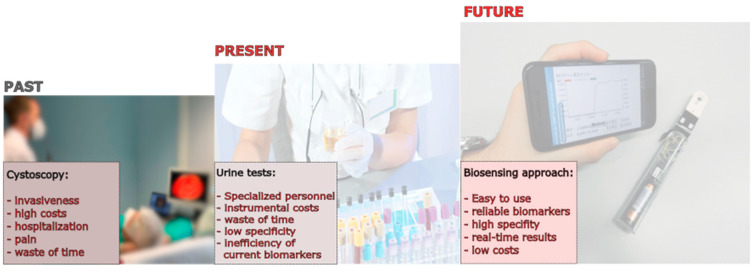
Past, present, and future perspectives in the management of bladder cancer.

BlCa is a diverse illness with intricate mechanisms that are difficult to treat quickly or effectively. In fact, one of the largest problems in BlCa management is improving diagnosis and follow-up to significantly boost survival rates. The development of biosensing tools for BlCa diagnosis and prognosis using urine samples represents a very important step towards the future management of BlCa (Figure 3). Having highly sensitive and non-invasive tools to permit population screening and surveillance of BlCa will not only improve quality of life and increase life expectancy (survival) but can also reduce the economic burden on the national health care systems. In addition, non-invasive and affordable diagnostic tools, like biosensors, will benefit people of lower SES who are more exposed to BlCa, reducing the inequality within and between populations of different countries.

## Figures and Tables

**Table 1:** Summary of potential BlCa diagnostic tests.

Test	Sensitivity	Specificity	Reference
Microsatellite analysis	72–97%	80–100%	[22]
Analysis EV	81%	90%	[23]
EpiCheck	94.3%	79.6%	[24]
BTA STAT	57–82%	68–93%	[25]
BTA TRAK	66–77%	5–75%	[26]
CellDetect	94%	89%	[27]
CxBladder	82%	90%	[28]
ImmunoCyt (uCyt+)	60–100%	75–84%	[29]
NMP22 BladderChek (ELISA)	69%	77%	[25]
NMP22 BladderChek (POC)	58%	88%	[25]
UBC	64.4%	80.3%	[25]
URO17	100%	96%	[30]
UroVysion	69–87%	89–96%	[31]

## Data Availability

No new data were created as part of this paper.
